# Could Daily Monitoring of Fibrin Related Markers Help Suspect a Thrombotic Event in COVID-19 Patients? A Prospective Pilot Study

**DOI:** 10.1055/s-0041-1728722

**Published:** 2021-05-12

**Authors:** Michael Hardy, Isabelle Michaux, Alain Dive, Thomas Lecompte, François Mullier

**Affiliations:** 1 Université catholique de Louvain, CHU UCL Namur, Namur Thrombosis and Hemostasis Center (NTHC), Namur Research Institute for Life Sciences (NARILIS), Hematology Laboratory, Yvoir, Belgium; 2 Université catholique de Louvain, CHU UCL Namur, Namur Thrombosis and Hemostasis Center (NTHC), Namur Research Institute for Life Sciences (NARILIS), Anesthesiology Department, Yvoir, Belgium; 3 Université catholique de Louvain, CHU UCL Namur, Intensive Care Department, Yvoir, Belgium; 4 Division of Angiology and Hemostasis - Geneva Platelet Group, Departement of Medicine, Université de Genève et Hôpitaux Universitaires de Genève, Geneva, Switzerland

Coronavirus disease 2019 (COVID-19) is associated with a high incidence of thrombotic events (TE). 1 Prompt recognition of thrombosis in COVID-19 patients is highly relevant. Signs heralding a TE can be very difficult to detect however, particularly in the intensive care setting. What a proper clinical suspicion of an acute VTE is in that setting remains elusive and documenting a pulmonary event can be a huge challenge for such severely affected, unstable patients. Therefore, the value of laboratory markers indicating fibrin formation in vivo in increasing the suspicion of an on-going TE deserves to be studied.

D-dimers are cross-linked fibrin degradation (plasmin) products containing the D-dimer motif. Their plasma levels indicate the risk for TE in COVID-19 patients. 2 3 4 5 6 Assays for D-dimers are widely and readily available, around the clock. Rises during hospital stay could point to the occurrence of TE. 6 However, D-dimers plasma levels are elevated in COVID-19 patients already on admission 6 7 ; therefore finding a clinically useful threshold to alert of a possible on-going TE is not easy. Fibrin monomers (FM) are other ‘fibrin-related markers’ (FRM), which differ from D-dimers, their formation not depending on fibrinolysis, which is impaired in COVID-19 patients. 8 9 As D-dimers, FM are easily measured in citrated samples and can rise in case of both arterial and venous thrombosis. 10 To the best of our knowledge, there are few available data on FM in COVID-19 patients. 11 12 We therefore undertook a prospective observational exploratory monocentre study to get some insight as to whether their serial monitoring might help suspect an on-going thrombosis.

Twenty-one consecutive patients admitted to the intensive care unit (ICU) of an academic hospital with reverse transcription–polymerase chain reaction confirmed COVID-19, all prophylactically administered with heparin, were included from March 27 to April 24, 2020. 8 Enoxaparin was the preferred anticoagulant. Unfractionated heparin (UFH) was used in case of extracorporeal oxygen membrane oxygenation (ECMO), renal failure or high bleeding risk. FRM (D-dimers and FM in soluble fibrin complexes – Liatests, Stago; normal FM values < 6μg/mL; FM limit of quantification 5μg/mL, even though reported measures are sometimes lower 13 ) were measured in double-centrifuged frozen-thawed citrated plasma samples, daily prepared. 8 Deep vein thrombosis (DVT) was diagnosed by ultrasound examination when clinically suspected and systematically once a week. Pulmonary thrombosis - embolism was diagnosed when clinically suspected by computed tomography angiography or by cardiac echography according to patient's status. D-dimers changes along the ICU stay and their individual patterns were already reported. 14

Ten patients were diagnosed with thrombosis, seven of them within eight days after ICU admission: five DVT (two symptomatic and three asymptomatic), two seemingly isolated PE, one patient with both, and two ischemic strokes. Five patients were under ECMO at some point of their ICU stay and one patient fulfilled ISTH overt disseminated intravascular coagulopathy criteria (DIC; both with D-dimers or FM as FRM 15 ) while also suffering from ischemic stroke.

**Fig. 1:**
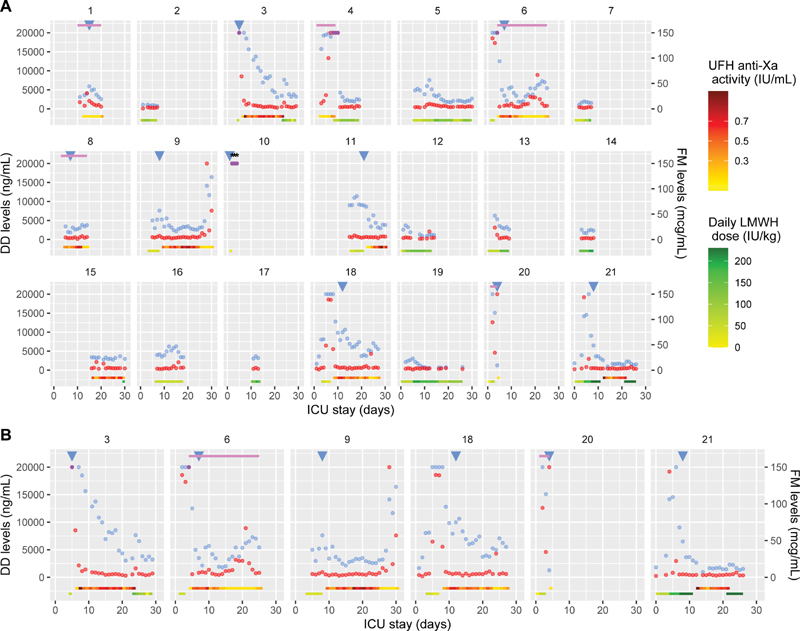
Daily changes of D-dimers and fibrin monomers (FM) plasma levels during the intensive care unit stay for the 21 study patients (A) and for the six patients with a FM peak (B). The late FRM peak in patient 9 followed radioembolization of gluteal bleeding, which may explain the increase in FRM. 19 D0 is the day of first ICU admission (11 patients were transferred from another ICU to ours). D-dimers levels are represented with blue dots and FM levels with red dots (purple dots correspond to superimposed values). Blue triangles correspond to days of thrombosis diagnosis and black stars to days when ISTH criteria for overt disseminated intravascular coagulopathy were met (either using D-dimers or FM as fibrin-related markers 15 ). Upper pink lines represent the period of extracorporeal membrane oxygenation treatment. Daily LMWH (enoxaparin) received dose (green line) and achieved anti-Xa levels for UFH administration (red line) are represented in the bottom of the figure. UFH, unfractionated heparin; LMWH, low molecular weight heparin; DD, D-dimers; FM, fibrin monomers.

Results of both FRM along with main clinical features are represented over time in Fig. 1 for each patient. The maximal FRM levels (medians) observed before censoring (TE, death, ICU discharge or end of study period) were higher in patients diagnosed with a TE than in patients who were not: 20,000 (IQR: 7,610–20,000) versus 3710 ng/mL (2,825–6,265) for D-dimers ( *p* = 0.009, Mann-Whitney U test); 139 (8–150) versus 10 μg/mL 4 5 6 7 8 9 10 11 12 13 14 15 16 for FM ( *p* = 0.05, Mann-Whitney U test). We arbitrarily defined peak levels as follows: above 15,000 ng/mL or 100 μg/mL for D-dimers and FM respectively. Out of nine TE patients (one being excluded because no FRM levels were available before diagnosis of thrombosis), for five peaks preceded thrombosis diagnosis; and for the other 11 patients, ten were without; this held true for both FRM. Thus FRM peaks could help deciding which patients deserve imaging investigations. Additional studies should however be performed to confirm this hypothesis, to determine appropriate thresholds and to refine the respective role of each FRM.

In this regard, it is interesting to note that FM levels were often within the manufacturer's reference range (i.e., median proportion of ICU stay with FM below 6μg/mL was 83% out of 299 measurements), in sharp contrast with very frequent high D-dimers. This argues against intravascular fibrin formation as the main contributor to D-dimers plasma levels, even in severe COVID-19 patients. The molecular mass of the former (basically one FM is associated with two fibrinogen molecules in a soluble complex: roughly a thousand kDa) is such that what is circulating in blood most likely comes from the vasculature. 16 By contrast, plasma D-dimers, which have lower masses than the former, could mainly come from extravascular deposits in the lungs. 4 5 D-dimers levels would thus be more dependent on alveolar inflammation and damage – and thus on disease severity – than on intravascular fibrin formation. 17 Of note, FM levels were consistently high in two out of the five ECMO patients, which could come from fibrin deposition on the oxygenator membrane.

The important finding that FM plasma levels are often low is advantageous to capture an abrupt rise. Moreover, FM plasma levels decreased more rapidly after the peak than D-dimers. Indeed, FM peak levels were transient, perhaps specifically capturing intravascular fibrin formation, whereas D-dimers levels remained high for a more prolonged time-lapse. This could be explained by the shorter plasma half-life of FM and their production being less dependent on the fibrinolysis as compared with D-dimers. 9

Our study has limitations: (i) few patients were studied (354 patient-days however, including 221 patient-days until censoring); (ii) regarding the time-course of FRM, some patients seemed to have been admitted at the onset of TE and a true FRM baseline level was therefore not available; (iii) precise determination of timing of TE is complicated; (iv) discrimination could be more difficult in case of DIC; only one patient fulfilled ISTH overt DIC criteria in the study, precluding any conclusion regarding this issue; (v) the course of FRM plasma levels is likely to be influenced by the anticoagulation regimen as well, further complicating the issue. 18

To conclude, both FRM seem able to capture an on-going TE in most patients. An abrupt elevation should comfort in the clinical decision making to document TE and to consider therapeutic anticoagulation. Since D-dimers are always elevated in critical patients, sometimes markedly, FM plasma levels could be more striking warnings for an ongoing TE over a background of frequent normal levels and our data nominate it a candidate biomarker to investigate. As FM peaks are only transient, close monitoring would be required; whether it should be daily or otherwise deserves to be studied.
